# Inhibitory Effects of Myriocin on Non-Enzymatic Glycation of Bovine Serum Albumin

**DOI:** 10.3390/molecules27206995

**Published:** 2022-10-18

**Authors:** Libo He, Yang Liu, Junling Xu, Jingjing Li, Guohua Cheng, Jiaxiu Cai, Jinye Dang, Meng Yu, Weiyan Wang, Wei Duan, Ke Liu

**Affiliations:** 1 Key Laboratory of Bio-Resource and Eco-Environment of Ministry of Education, College of Life Sciences, Sichuan University, Chengdu 610065, China; 2 Department of Central Laboratory, The First People’s Hospital of Huzhou, First Affiliated Hospital of Huzhou University, Huzhou 313000, China; 3 School of Medicine, Deakin University, Geelong, VIC 3216, Australia

**Keywords:** Myriocin, non-enzymatic glycation, BSA, spectroscopic techniques, computational simulations

## Abstract

Advanced glycation end products (AGEs) are the compounds produced by non-enzymatic glycation of proteins, which are involved in diabetic-related complications. To investigate the potential anti-glycation activity of Myriocin (Myr), a fungal metabolite of *Cordyceps*, the effect of Myr on the formation of AGEs resulted from the glycation of bovine serum albumin (BSA) and the interaction between Myr and BSA were studied by multiple spectroscopic techniques and computational simulations. We found that Myr inhibited the formation of AGEs at the end stage of glycation reaction and exhibited strong anti-fibrillation activity. Spectroscopic analysis revealed that Myr quenched the fluorescence of BSA in a static process, with the possible formation of a complex (approximate molar ratio of 1:1). The binding between BSA and Myr mainly depended on van der Waals interaction, hydrophobic interactions and hydrogen bond. The synchronous fluorescence and UV-visible (UV-*vis*) spectra results indicated that the conformation of BSA altered in the presence of Myr. The fluorescent probe displacement experiments and molecular docking suggested that Myr primarily bound to binding site 1 (subdomain IIA) of BSA. These findings demonstrate that Myr is a potential anti-glycation agent and provide a theoretical basis for the further functional research of Myr in the prevention and treatment of AGEs-related diseases.

## 1. Introduction

The non-enzymatic glycation is characterized by a series of reactions that occur between amino groups of protein, lipid, or nucleic acid and aldehyde groups of reducing sugars under non-enzymatic conditions. Finally, brown advanced glycation end-products (AGEs) with high fluorescence, cross-linking and irreversibility are formed [1,2]. The enhanced non-enzymatic glycation of many important proteins in high glucose environments is the main cause of late complications of diabetes, such as diabetic cardiovascular disease and diabetic nephropathy, etc. [3,4]. In particular, AGEs can elevate blood glucose levels [5], damage the wall of blood vessels [6,7], and induce obesity-associated insulin resistance [8,9]. Therefore, blocking glycation cascades has been a promising strategy for the prevention or treatment of diabetes and other pathogenic complications [10].

Serum albumin, the most abundant carrier protein in plasma [11], is responsible for transporting metabolites and drugs and maintaining osmotic pressure [12]. It is sensitive to non-enzymatic glycation [13,14]. The interaction between drugs and serum albumin affects the absorption, metabolism and excretion of drugs. Bovine serum albumin (BSA), consisting of 581 amino acid residues, is often selected as a model protein for non-enzymatic glycation reaction due to its wide availability and known structure information [11,15]. Albumin glycated by glucose changes its conformation and binding properties and triggers the downstream signaling pathway mediated by the receptor for AGEs (RAGE) [16,17].

Recently, natural compounds inhibiting non-enzymatic glycation reactions have attracted wide attention. For example, coumarin, oleanolic acid, silybin, ferulic acid, resveratrol and curcumin have been demonstrated to show effective anti-glycation activity and were expected to be potential drugs for diabetes and its complications [18,19,20,21].

Myriocin (Myr), isolated from fungi traditionally used in Chinese medicine, including *Isaria sinclairi*, *Myriococcum albomyces* and *Mycelia sterilia* [22], is a potent and highly selective serine palmitoyltransferase (SPT) competitive inhibitor with a molecular structure similar to the substrates of SPT [23]. It was reported that Myr has multiple biological activities, such as anti-tumor [24], ameliorating atherosclerosis [25,26,27,28,29], improving non-alcoholic fatty liver [30,31], preventing hepatic steatosis [32,33,34], hypertension [35], and cardiomyopathy, etc. [36,37]. In addition, research about diabetes mice has revealed that Myr can regulate insulin sensitivity, glucose tolerance, mitochondrial function and blood glucose level of obese mice, indicating that Myr can relieve the pathology of diabetes and its complications by reducing blood glucose level [38]. However, whether Myr inhibits the non-enzymatic glycation of proteins is still unclear.

In this study, we investigated the inhibitory capability of Myr on the protein glycation and the related mechanism by using a BSA-glucose model of non-enzymatic glycation. This study will provide elaborate information about the Myr-BSA binding phenomena and give insight into the development of Myr as an antiglycation agent.

## 2. Results and Discussion

### 2.1. Inhibitory Effect of Myr on Non-Enzymatic BSA Glycation

**Figure 1:**
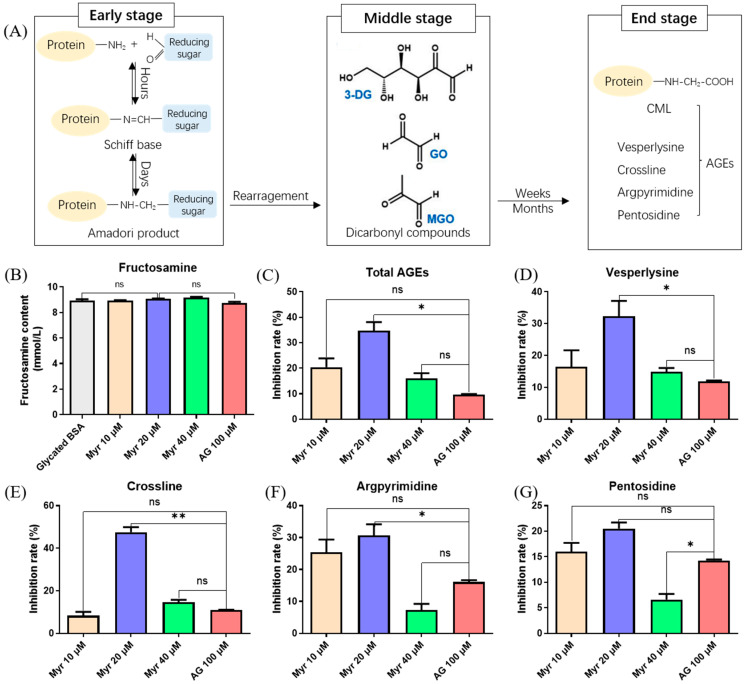
Effects of Myr on the glycation products formed in BSA-Glucose model at different stages. (**A**) Diagram of formation process of advanced glycation end products (AGEs). (**B**) Content of fructosamine generated at the early stage in BSA-glucose system incubated with Myriocin (Myr) or aminoguanidine (AG), measured by UV absorbance at 530 nm. (**C**) Inhibition rate of total AGEs generated at the end stage, measured by fluorescence emission at 440 nm with excitation at 350 nm. (**D**–**G**) Inhibition rates of different fluorescent AGEs including vesperlysine, crossline, argpyrimidine and pentosidine, measured by fluorescence at *λ_ex_/λ_em_* of 350/405, 380/440, 320/380 and 335/385 nm, respectively. Data were expressed as mean ± SD (n = 3). ns > 0.05, * *p* < 0.05 and ** *p* < 0.01 compared with AG.

The non-enzymatic glycation of proteins to form AGEs can be divided into three stages [39]. At the early stage, the reducing sugar is condensed with the free amino group of the protein to form a Schiff base, which then produces the Amadori product (Figure 1A). At the middle stage, the Amadori product further reacts with dicarbonyl compounds such as 3-deoxyglucosone (3-DG), glyoxal (GO) and methylglyoxal (MGO). At the end stage, the dicarbonyl compound reacts with the amino group to produce AGEs (Vesperlysine, Crossline, Argpyrimidine and Pentosidine) with fluorescent properties. The early glycation product, fructosamine, was analyzed by detecting the chromogenic product at 530 nm. Aminoguanidine (AG), a chemically synthesized compound that inhibits the formation of AGEs, was applied as positive control. Myr did not inhibit the formation of fructosamine (Figure 1B). However, Myr showed a strong inhibitory effect on the end glycation products. To be specific, 20 μM Myr inhibited 34% of total AGEs (Figure 1C), 32% of Vesperlysine (Figure 1D), 47% of Crossline (Figure 1E), 30% of Argpyrimidine (Figure 1F) and 20% of Pentosidine (Figure 1G), while AG only inhibited about 10% of these end glycation products. These results proved that Myr can delay the glycation reaction, especially at the end stage.

Myr, like AG, did not reduce the content of fructosamine, but inhibited the formation of end products, which was consistent with the previous study [40]. We speculate that this might be because Myr and AG have similar structures in terms of amino group, which has a low *K_a_* value and is easy to condense with the carbonyl group [41], so that they have strong competition in protein glycation reaction, thus preventing the generation of end products.

### 2.2. Anti-Fibrillation Ability of Myr

**Figure 2:**
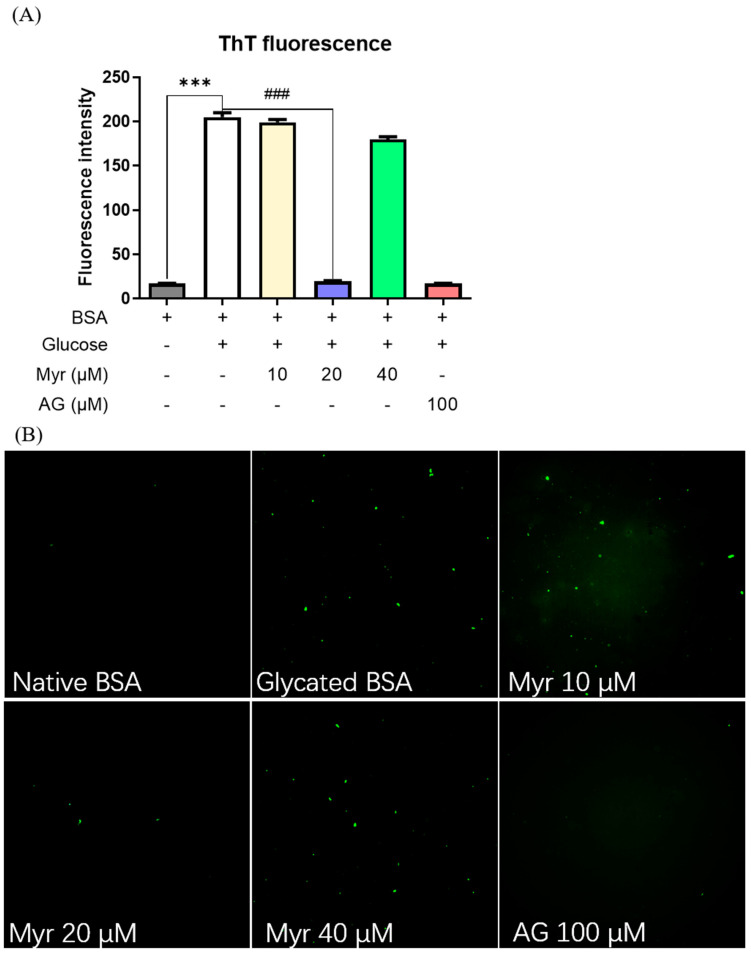
Effects of Myr on glycation-induced protein amyloid aggregation. (**A**) ThT fluorescence spectrometry analysis of amyloid-like fibrils in the presence and absence of Myr or AG (aminoguanidine, positive control). (**B**) The fluorescence microscopy analysis of amyloid-like fibrils in the absence and presence of Myr. *** *p* < 0.001 compared with native BSA. ^###^ *p* < 0.001 compared with glycated BSA.

Amyloid-like fibrils, formed by proteins crosslinking triggered by AGEs at the end stage of glycation [42], have been strongly related to some diabetic complications such as Parkinson’s disease and Alzheimer’s disease [43]. To monitor amyloid-like fibrils, BSA was stained with the fluorescent dye thioflavin T (ThT). ThT can bind to the β-folded structure of the protein and has strong fluorescence at 480 nm. The higher ThT fluorescence intensity of glycated BSA than native BSA indicated that protein amyloid fibrosis aggregation had formed (Figure 2A). With the addition of Myr, the fluorescence intensity was decreased, indicating that Myr could inhibit the amyloid aggregation of glycated protein. Similarly, the glycated BSA exhibited more fluorescence spots than native BSA (Figure 2B), indicating that amyloid-like fibrils were formed due to the glycation of BSA, while 20 μM Myr significantly decreased the number of fluorescence spots and is comparable to AG. These results indicated that Myr could prevent the crosslinking of BSA and avoid the formation of amyloid-like fibrils, which was consistent with the fluorescence intensity result.

### 2.3. Inhibitory Effect of Myr on Protein Glycoxidation

**Figure 3:**
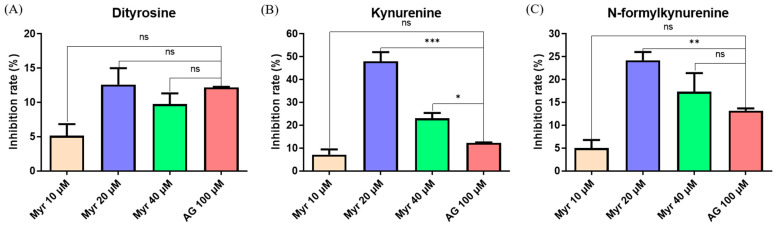
Effects of Myr on glycoxidation fluorescence products. Inhibition rates of formation of dityrosine (**A**), kynurenine (**B**) and N′-formyl-kynurenine (**C**), respectively, measured by fluorescence at 415, 480, and 434 nm with excitation at 330, 365, and 325 nm, respectively. Data were expressed as mean ± SD (n = 3). ns > 0.05, * *p* < 0.05, ** *p* < 0.01 and *** *p* < 0.001 compared with AG.

Non-enzymatic glycation is commonly accompanied by an oxidation process called glycoxidation [44]. The tryptophan (Trp) and tyrosine (Tyr) residues of proteins can be destroyed by glycoxidation, which results in different fluorescent products. Trp residues are easily modified to Dityrosine and N′-formylkynurenine, while Tyr residues are easily modified to Kynurenine. Therefore, we monitored the formation of these fluorescent products during the glycoxidation of BSA by spectrometry. We found that 20 μM Myr inhibited 10–15% of Dityrosine, which is similar to the activity of 100 μM AG (Figure 3A). Moreover, 20 μM Myr resulted in 48% and 24% inhibition of Kynurenine (Figure 3B) and N′-formylkynurenine (Figure 3C), respectively, and the inhibitory ability of Myr on the glycoxidation products was stronger than AG. These results revealed that Myr protected the Trp and Tyr residues of BSA from glycoxidation.

### 2.4. Quenching Effect of Myr on Endogenous Fluorescence of BSA

**Figure 4:**
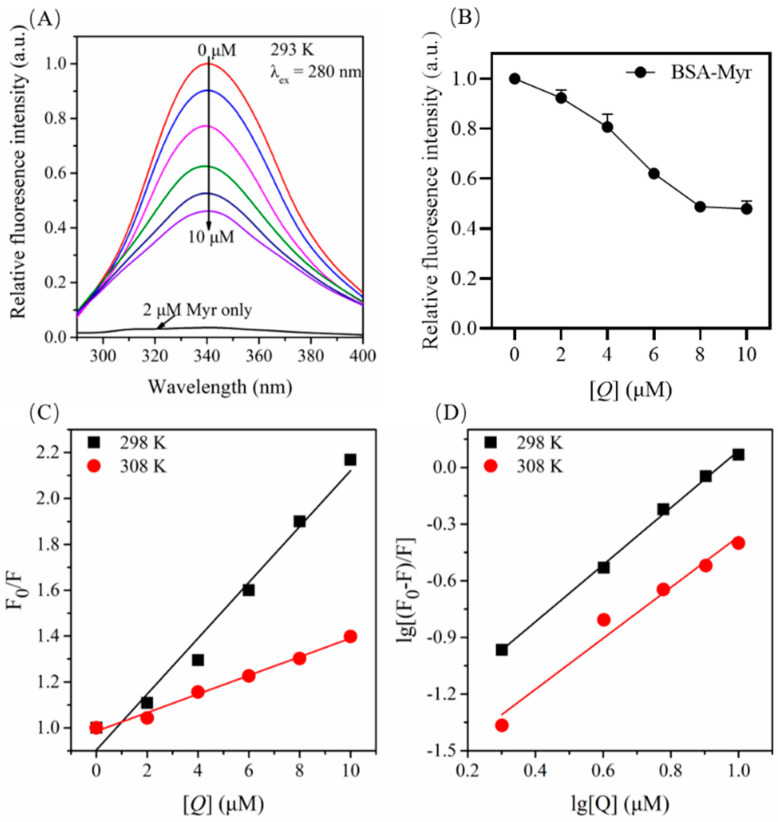
Fluorescence emission spectra of Myr-BSA system in 10 mM PBS (pH = 7.4) at 293 K. (**A**) Fluorescence emission spectra for BSA (2 μM) with Myr (0, 2, 4, 6, 8, 10 μM). (**B**) The fluorescence intensity of BSA at 340 nm in absence and presence of Myr (0–10 μM). (**C**) The *Stern*-*Volmer* plots of fluorescence quenching constant (*K_q_*) for the Myr-BSA complexes at different temperatures. (**D**) The double logarithmic plots at different temperatures.

The phenomenon of decreasing fluorescence intensity after interaction between protein and small molecules is called fluorescence quenching. Fluorescence quenching of the protein upon drug binding was used to find the drug–protein interactions and measure the binding affinity between drug and protein [45]. To understand the interaction mechanism between Myr and BSA, we carried out the fluorescence quenching experiment. When the excitation wavelength is 280 nm, the peak of endogenous fluorescence emitted by BSA is about 340 nm, while Myr itself had almost no fluorescence emission peak at this excitation wavelength (Figure 4A). The maximum fluorescence (340 nm) of BSA was quenched with the increasing concentrations of Myr (Figure 4B), but the peak position and waveform did not change significantly. This indicated that Myr retards the glycation of BSA by glucose since Myr interacts with BSA.

### 2.5. Fluorescence Quenching Mechanism for BSA by Myr

The fluorescence quenching mechanism of proteins can be divided into static quenching, dynamic quenching, and static combined dynamic quenching [46]. Static quenching refers to the combination of a protein and a small molecule ligand to form a ground state complex, which is accompanied by the quenching of the endogenous fluorescence of a protein, while dynamic quenching is the result of the collision of a protein and a small molecule ligand with no change in the fluorescence spectrum. Generally, the types of quenching mechanisms can be distinguished by their difference in temperature dependence. The fluorescence quenching mechanism can be determined according to the *Stern–Volmer* equation [47]:

*F*_0_*/F* = 1 + *K_q_τ*0*[Q]* = 1 + *K_sv_ [Q]* (1)

where *F*_0_ and *F* are the fluorescence intensity of BSA in the absence and presence of Myr, respectively. *[Q]* is the concentration of Myr. *K_q_* and *K_sv_* are the bimolecular quenching rate constant and *Stern–Volmer* quenching constant, respectively. *τ*0 is the mean lifetime of fluorescent molecules (about 10^−8^ s) [48].

The *Stern–Volmer* curve shows a linear relationship (Figure 4C), indicating that the binding of Myr and BSA involves a single quenching mechanism. In addition, the slope of the fitting curve decreased with the increase in temperature, which was consistent with the characteristics of static quenching. As shown in Table 1, with the increase in temperature, *K_sv_* of the system gradually decreased and was 2–3 orders of magnitude larger than the maximum dynamic quenching rate constant of 100 mol/L for biological macromolecules. Consequently, the fluorescence quenching of BSA by Myr was a static quenching process. The bimolecular quenching rate constant *K_q_* (1.21 × 10^13^) was much larger than the maximum of the dynamic collision quenching constant 2.0 × 10^10^ Lmol^−1^ s^−1^ [49], further confirming that the fluorescence quenching mode of BSA by Myr was static quenching.

### 2.6. Binding Constants and Number of Binding Site for the BSA-Myr Complex

Fluorescence intensity in static quenching follows Equation (2) [50,51]:

lg[(*F*_0_ − *F*)/*F*] = lg*K_a_* + *n*lg[*Q*] (2)

where *K_a_* is the binding constant, which is commonly used to represent the binding strength of protein and small molecule. *n* is the number of binding sites, indicating the ratio of the number of binding sites between small molecules and protein.

lg[(*F_0_* − *F*)/*F*] is plotted against lg[*Q*], and the results are shown in Figure 4D. The number of binding sites n and the binding constant *K* of Myr and BSA can be calculated from the slope and intercept of the line (Table 1). The binding constant *K_a_* was 3.79 × 10^4^ mol/L at 298 K, which is larger than the binding constant of general ligand–protein interactions, indicating that Myr had a strong affinity with BSA. When the temperature rose to 308 K, *K_a_* decreased by nearly 2 times (1.93 × 10^4^ mol/L), validating that the decomposition of the Myr-BSA complex occurred at higher temperature. The above results ulteriorly confirmed that the interaction mode of Myr-BSA was static binding. In addition, the number of binding sites (n) between Myr and BSA at 298 K and 308 K were 1.5 and 1.35, respectively, which were both close to 1, suggesting that BSA interacts with Myr in a one-to-one ratio. Thus, we speculated that there may be only one binding site between Myr and BSA.

### 2.7. Binding Forces between BSA and Myr

In general, the binding force between ligands and proteins mainly involves the following four non-covalent interactions: hydrogen bonding, van der Waals forces, electrostatic interactions and hydrophobic interactions [52]. The thermodynamic parameters such as enthalpy change (*ΔH*), drip change (*ΔS*), and Gibbs free energy change (*ΔG*) were calculated using Equations (3) and (4) [53]:

*ln K_a_ = −ΔH/RT + ΔS/R* (3)

*ΔG = ΔH − TΔS* (4)

where *K_a_ is* the binding constant of small molecules to proteins. *T* and *R* represent temperature and the gas constant, respectively.

*ΔH* is ignored when the temperature varies within a small range. The relationship between thermodynamic parameters and force type of small molecules-protein interaction can be determined as follows: *ΔH* > 0, *ΔS* > 0 correspond to hydrophobic forces; *ΔH* < 0, *ΔS* < 0 correspond to van der Waals interaction, hydrogen bond formation; *ΔH* < 0, *ΔS* > 0 correspond to electrostatic interactions force [54]. *ΔH* and *ΔS* were both negative values (Table 1) indicated the force types were van der Waals interaction and hydrogen bond between Myr and BSA. Additionally, the negative values of *ΔG* indicated that the interaction between BSA and Myr is spontaneous [55].

### 2.8. Influence of Myr on the Structure of BSA as Monitored by Synchronous Fluorescence Spectroscopy

**Figure 5:**
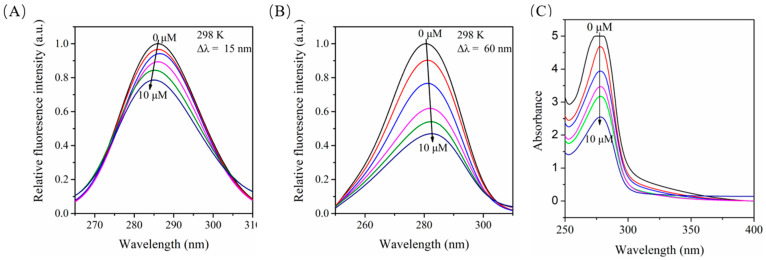
Effects of Myr on the conformation of BSA. (**A**,**B**) represent the synchronous fluorescence spectra of the interaction between Myr and BSA at *Δλ* = 15 nm and *Δλ* = 60 nm, respectively. (**C**) UV-*vis* absorption spectra of BSA in absence and presence of Myr (0, 2, 4, 6, 8, 10 μM).

Synchronous fluorescence spectrum is used to evaluate the changes of the microenvironment around Tyr and Trp residues. The overlapping fluorescence peaks in the ordinary fluorescence spectrum can be separated by selecting the appropriate wavelength difference. When *Δλ* = 15 nm, only the fluorescence of Tyr residue was observed, and when *Δλ* = 60 nm, the fluorescence of Trp residue was observed [56]. Since the maximum emission wavelength of fluorescent amino acid residues is related to the polarity of the environment [57], changes in protein conformation can be determined by analyzing the results generated by changing the emission wavelength. We fixed the concentration of BSA, gradually increased the concentration of Myr, and scanned the synchronous fluorescence spectra of BSA when *Δλ* = 15 nm and *Δλ* = 60 nm. With the increase in concentration of Myr, the fluorescence of both Tyr and Trp residues in BSA were quenched, and the fluorescence quenching degree of Trp residues was greater than that of Tyr residues (Figure 5A,B), clarifying that the binding site between Myr and BSA was closer to the Trp residues. Simultaneously, the maximum emission wavelength of Tyr residues and Trp residues in BSA showed a slight blue shift (Figure 5A) and red shift (Figure 5B), respectively. We speculate that this might be due to Myr enhancing the polarity near the Trp residues in BSA, weakening the hydrophobic environment, and increasing the extent of the peptide chain extension. All of these results suggest that Myr changed the conformation of BSA.

### 2.9. Effects of Myr on the UV-Vis Absorption of BSA

UV-*vis* absorption spectrometry is a simple and efficient method to study protein conformational changes and complex formation. The intensity of the absorption peak of BSA at 280 nm decreased significantly with the increase in concentration of Myr (Figure 5C). These results indicate that the interaction between Myr and BSA can lead to the conformation change of BSA, and also confirmed that Myr binds to BSA to form the ground state complex; that is, the fluorescence quenching mechanism between them is static quenching.

### 2.10. Binding Model between BSA and Myr

Studies have shown that small molecules have three binding sites on BSA: site 1 (subdomain IIA) is the warfarin (War) binding site, site 2 (subdomain IIIA) is the ibuprofen (Ibu) binding site [58], and site 3 is the methyl orange (Met) binding site [59]. Each binding site contains two subdomains. In order to further determine the specific binding site of Myr on BSA, Met, War and Ibu were used to carry out locus competitive labeling experiments. The substitution percentage of site labeled probes can be calculated by the following equation [60]:

(5) $$\mathrm{Probe}\mathrm{displacement}(\%)=\frac{F_{2}}{F_{1}}\;\times \;100\%$$

where *F*_2_ and *F*_1_ are the fluorescence intensity of BSA-Myr complex in the presence and absence of site labeled probe, respectively.

**Figure 6:**
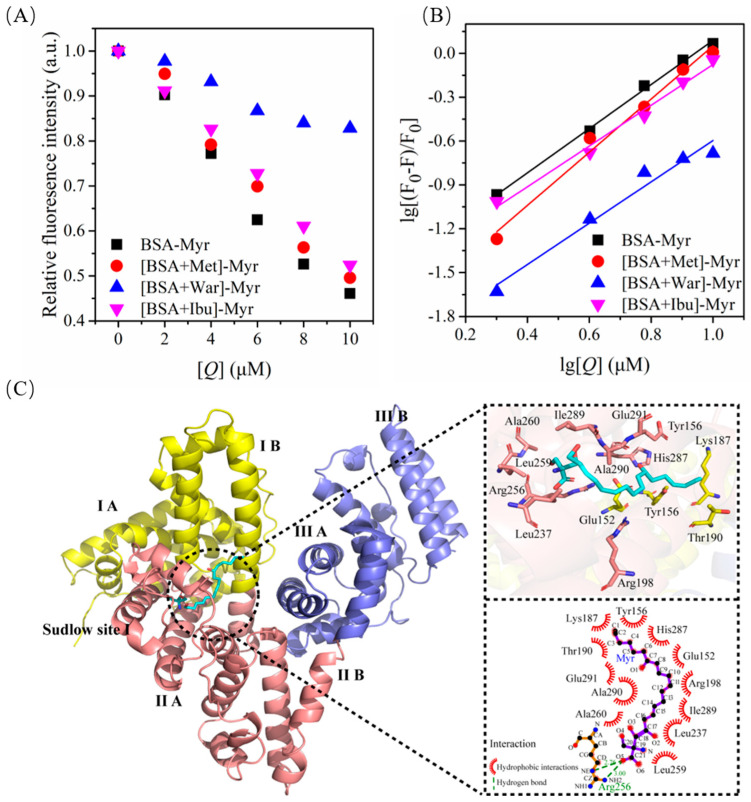
The binding model between BSA and Myr. (**A**) Fluorescence intensity of BSA-Myr in presence of Met, War and Ibu; pH = 7.0, T = 298K, *λ_ex_* = 280 nm. (**B**) Binding constant plots of BSA-Myr in presence of Met, War and Ibu; *λ_ex_* = 280 nm. (**C**) The lowest binding free energy pose for Myr into the BSA site I: 3D view cartoon presentation (**left**) and the 2D schematic view of the binding interaction (**right**).

The relative fluorescence intensity of BSA-Myr system decreased slowly in the presence of War, but remained decreased in the presence of Met and Ibu (Figure 6A). In other words, Myr could not reduce the fluorescence intensity of BSA after site 1 was competitively bound by War. Figure 6B represents the plot of binding constant for the BSA-Myr system in the presence of War, Met and Ibu. Consistently, as listed in Table 2, the considerable decrease in binding constant *K’_a_* value for the [BSA + War]-Myr system (0.98 × 10^4^ mol/L) compared to the BSA-Myr system (3.79 × 10^4^ mol/L) signifies a similarity of binding site between War and Myr to BSA. The basically unaltered *K’_a_* value of BSA-Myr system in the presence of Ibu indicated the low affinity of Myr towards site 2. These findings revealed that the main binding site between Myr and BSA was site 1 (subdomain IIA).

Next, the possible binding mode of Myr on the three-dimensional structure of BSA was further studied by molecular docking simulation. The best conformation of Myr was selected with the binding energy of −6.5 kcal/mol. Myr docked into the subdomain IIA cavity of BSA to form the Myr-BSA complex, which was mainly surrounded by Leu237, Arg256, Leu259, Ala260, Ile289, Ala290, Glu152, Glu291, His287, Lys187, Tyr156, Thr190, and Arg198 (Figure 6C). This was consistent with the binding site of most small molecule drugs being site 1 [61,62]. Furthermore, molecular docking revealed that hydrogen bonding and hydrophobic interactions existed between Myr and BSA, which was consistent with the results of thermodynamic parameters mentioned above (Section 2.7). The oxygen atom from the carboxyl group at C-21 of Myr formed two hydrogen bonds with Arg 256 of BSA with the distances of 2.76 and 3.00 Å, respectively. Additionally, Lys and Arg were reported to be the pivotal amino acids responsible for BSA glycation [63,64,65]. In this study, Myr could bind to BSA through hydrogen bonding and hydrophobic interaction with several Lys and Arg residues of BSA, which further proved that Myr could prevent the glycated reaction of BSA from sugars.

### 2.11. Molecular Dynamics (MD) Simulation between BSA and Myr

**Figure 7:**
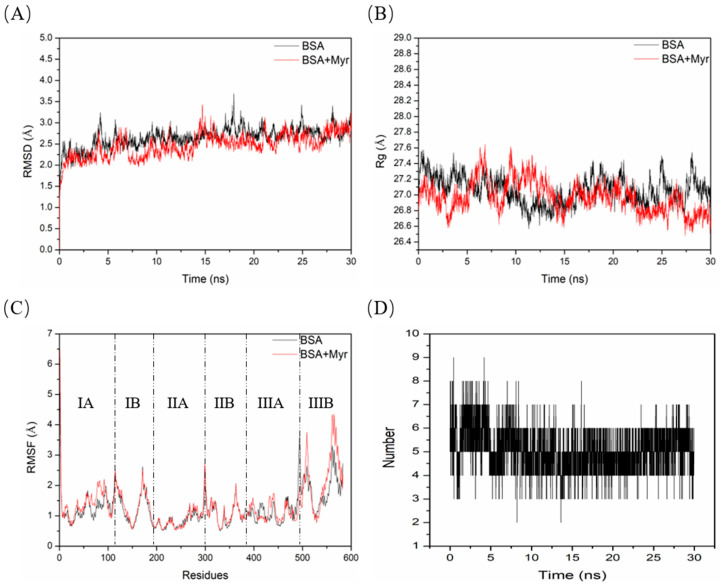
The results of 30 ns MD simulations. (**A**–**C**) represent the RMSD, Rg and RMSF values of the BSA and BSA-Myr systems, respectively. (**D**) Hydrogen bonds involved during the interaction between Myr and binding pocket residues of BSA.

To assess the binding stability of the BSA-Myr system at the atomic level, 30 ns MD simulations were performed. MD simulation allows the observation of structural information of complex systems over time at the atomic level [66,67]. Root mean square deviation (RMSD) is an essential index for evaluating the structural stability in MD simulation [68,69]. BSA and BSA-Myr systems reached relative equilibrium after 5 ns when running a MD simulation with an average backbone C_α_-RMSD of about 3 Å (Figure 7A). The radius of gyration (Rg) can be used to estimate the density of the target protein. Thereby, the increase in Rg value reflects the decrease in the density and loose structure of the target protein [67,70]. The Rg value of BSA-Myr system was slightly higher than that of the BSA system in 30 ns MD simulation (Figure 7B), indicating that Myr bound to BSA, resulting in its density reduction and loose structure. Root mean square fluctuation (RMSF) is a crucial index for evaluating the flexibility of amino acid residues in MD simulation [71]. We observed that the RMSF value of the residues 400–420 of the BSA-Myr system is slightly lower than that of BSA (Figure 7C), illuminating that these residues are potentially key binding residues. This result is partially different from molecular docking, which may be the dynamic binding process during the binding of Myr to BSA. By analyzing the change of hydrogen bond in complex system, we can not only study the bond stability of complex systems, but also determine the bond strength of complex systems [72]. The average number of hydrogen bonds in BSA-Myr systems was five during 30 ns MD simulation (Figure 7D), which means Myr bound to the key residues in BSA via hydrogen bonding to maintain the stability of the complex system.

A distinct feature of our study is that Myr exhibited antiglycation and anti-glycoxidation potential. The reason why Myr could prevent the glycation of BSA from sugars is that Myr bound to site 1 of BSA through hydrogen bonding and hydrophobic interaction with several Lys and Arg residues, which are involved in BSA glycation. Although the antiglycation mechanism of Myr is not completely understood, it is certain that Myr is hydrophobic and forms complexes with hydrophobic domains of BSA, which could play a critical role in attenuating the glycation and associated changes in conformation of BSA. As a result, this work provides novel insights into the mechanism of antiglycation properties of Myr, which can form the basis of future antiglycation strategies in both diabetic and non-diabetic individuals. However, the limitations of the present studies include (1) a lack of determination about inhibition of intermediate glycation products, and that (2) only the structural information of the microenvironment surrounding the endogenous fluorophores of BSA was monitored. Circular dichroism spectroscopy needs to be carried out in the future to verify the secondary structural conformational changes in BSA.

AGEs accumulation is an important factor in metabolic disorders. It has been demonstrated that glycated serum albumin [73] and glycated lipoproteins levels [74] are higher in diabetic patients than healthy subjects. Glycated albumin participates in the pathogenesis of diabetic nephropathy by increasing the ROS accumulation and activating inflammation [75,76]. Glycated lipoproteins aggravate the pathological process of atherosclerosis [77]. The inhibition of AGEs formation is one of the targets for the treatment of these metabolic disorders. In this study, we revealed that Myr can interact with BSA (Figure 6) to inhibit the formation of AGEs (Figure 1, Figure 2 and Figure 3), which provides new insight into the mechanism of Myr against the above metabolic disorders.

## 3. Materials and Methods

### 3.1. Regents

Myriocin (APEx, Houston, TX, USA, 98%, #B6064), aminoguanidine (Sigma-Aldrich, St. Louis, MI, USA, 98%, #396494) and BSA (Sigma-Aldrich, USA, 98%, #A1933) were prepared as 2 mM, 1 mM and 0.1 mM stock solution in 10 mM PBS, respectively. D-Glucose was purchased from Chronchem Co., Ltd. (Chengdu, China). Methyl orange (Met), warfarin (War) and ibuprofen (Ibu) and thioflavin T (ThT) were all purchased from Yuanye Bio-Technology (Shanghai, China). All reagents used were analytical grade. Ultrapure water was used throughout the experiments. Stock solutions were prepared weekly and stored in the dark at 4 °C.

### 3.2. Non-Enzymatic Glycation of BSA

AGEs were prepared by co-incubation of glucose and BSA, as described previously [78]. Briefly, BSA solution (50 mg/mL in PBS buffer) was incubated with or without D-glucose (0.5 M) for 90 days at 37 °C followed by dialyzing in PBS (pH 7.4, 5 mM) at 4 °C overnight. The incubated solutions were then used for the following assays.

### 3.3. Fluorescence Spectroscopy

Fluorescence spectra were recorded using a Hitachi F-4500 fluorescence spectrophotometer (Hitachi, Kyoto, Japan) equipped with a water bath at 25 °C as described before [79]. We measured the fluorescence emission spectra (290–500 nm) of BSA (2 μM) with or without Myr (0–40 μM) with a scan speed of 1000 nm/min at 298 K and 308 K, respectively. Excitation and emission bandwidths were both 10 nm. PBS was used as a blank.

### 3.4. Anti-Glycation Activity of Myr

BSA (50 mg/mL in 10 mM PBS, pH 7.4) and different concentrations of sample solution were pre–mixed at room temperature for 30 min and then were added with glucose (0.5 M). The mixture was incubated in a water bath at 50 °C for 24 h. Ultimately, samples were removed from the water bath and tested.

#### 3.4.1. Determination of Fructosamine Content

We determined the content of early glycation product—fructosamine through nitroblue tetrazolium (NBT) method as described previously [80]. Briefly, the glycated protein sample (80 μL) was mixed with 1.6 mL NBT (0.3 mM, dissolved in 0.1 M sodium carbonate buffer, pH 10.35) in 320 μL water and incubated in 37 °C water bath for 15 min. Then, the absorbance at 530 nm was measured by the microplate reader. The inhibition rate of fructosamine was calculated by the following equation [71]:

The inhibition rate (%) = (1 − *A*_0_/*A*) × 100% (6)

where *A* and *A*_0_ are the absorbance of BSA with or without Myr (or AG), respectively.

#### 3.4.2. Determination of Fluorescent AGEs

The fluorescence intensity was measured as described in Section 3.3. Table 3 lists the excitation and emission wavelength of different AGEs [72]. After detection, the inhibition rate of fluorescent AGEs was calculated by the following equation [71]:

The inhibition rate (%) = (1 − *F*/*F*_0_) × 100% (7)

where *F* and *F*_0_ are the fluorescence intensity of BSA with or without Myr (or AG), respectively.

### 3.5. Detection of Amyloid-like Fibrils

The amyloid-like fibrils level was determined via thioflavin T (ThT) assay [81]. Briefly, native BSA, glycated BSA with or without Myr (or AG) were incubated with thioflavin T (300 μM) for 30 min at 25 °C in the dark, respectively. On the one hand, we measured the fluorescence intensity under *λ_ex_/λ_em_* of 440/485 nm using the method described in Section 3.3. On the other hand, the amyloid cross—β structure was observed using the fluorescence microscope (Leica DMi 8, Leica Microsystems, Wetzlar, Germany) [71,82].

### 3.6. Inhibitory Activity of Myr on Glycoxidation Fluorescence Products

Glycoxidation accompanied by glycation produces fluorescent products involving Dityrosine, kynurenine and N′-formylkynurenine [71]. We measured their fluorescence intensity under *λ_ex_/λ_em_* of 330/415, 365/480 and 325/434 nm using method described in Section 3.4, respectively. The inhibition rate of glycoxidation products was calculated by the same equation in Section 3.4.2.

### 3.7. UV-Visible Absorption Spectroscopy

UV-*vis* absorption spectra were recorded using a UV—2550 UV/*vis* spectrophotometer with 1.0 cm optical path quartz cuvette. Spectral measurements of BSA (2 μM) in the absence and presence of Myr (0–10 μM) were made in the range of 250–400 nm at 298 K. Corresponding spectra of Myr at different concentrations were recorded at the same condition and subtracted from the sample solutions.

### 3.8. Probe Displacement Experiment

To characterize the binding site between Myr and BSA, warfarin (site 1), ibuprofen (site 2), and methyl orange (site 3) at molar ratios of 1:0 and 1:1 were titrated against the complexes [59] of BSA (2 μM) and Myr (0–10 μM). The fluorescence intensity under 280 nm was determined using the method described in Section 3.3.

### 3.9. Molecular Docking

Molecular docking of Myr to BSA was carried out by AutoDock software (v4.2.6; Ruth Huey; La Jolla, CA, USA. http://autodock.scripps.edu/faqs-help/tutorial (accessed on 25 May 2020)) [83]. The structure of Myr was obtained from the ZINC database (http://zinc.docking.org/ (accessed on 25 May 2020)), followed by energy minimization using Chimera (v1.16; UCSF Resource for Biocomputing Visualization and Informatics; Topeka, KS, USA) [84,85]. The crystal structure of BSA (PDB_ID: 4F5S) was downloaded from the RSCB website (http://www1.rcsb.org/ (accessed on 25 May 2020)). Then, the water molecules, heteroatoms, ions, co-crystallized ligands and repeated chains were removed using PyMOL software (v2.0; Schrödinger; Shanghai, China) and the missing residues were completed using SWISS-MODELING software (Computational Structural Biology Group; Basel, Swiss. https://swissmodel.expasy.org (accessed on 25 May 2020)) [86]. Then, the docking was conducted in the grid box of 120 × 120 × 120 points with a grid space of 0.375 Å. Additionally, we calculated the possible binding conformation of Myr–BSA via the lamarckian genetic algorithm [87] and selected the lowest binding energy and highest score direction [79].

### 3.10. Molecular Dynamics (MD) Simulation

MD simulation of BSA and BSA–Myr complex were performed on Gromacs v4.6.5 tools [88]. Based on the docking result, the binding model of the BSA–Myr complex was selected as the initial conformation for MD simulation. The topology files of BSA were obtained by using the *pdb2gmx*. The topology files of Myr were generated by the antechamber and tleap tools from Ambertools v18 software using the AM1/BCC charge method [89]. The force field of BSA and Myr were set to Amberff99SB and GAFF (a general Amber force field), respectively [89]. The BSA and BSA–Myr systems were preprocessed by the following steps: (1) the TIP3P water molecules were inserted into the systems with the dodecahedron box; (2) Na^+^ and Cl^−^ were added to neutralize the systems; (3) the periodic boundary condition with a minimal distance of 1.2 nm was set between the complex and the surface of the box [90]. The systems first had their energy minimized using the steepest descent minimization method to limit the energy to 1000 Kcal/mol/nm [89]. Before the formal simulation, the complex system was carried out at both 100 ps NVT and NPT ensemble with 300 K temperature and 1 atm pressure [91,92]. Finally, 30 ns MD simulations of the BSA and BSA–Myr systems were run on Gromacs v4.6.5 programs with a time step of 2 fs. Additionally, the PME (Particle Mesh Ewald), LINCS (Linear Constraint Solver), and SETTLE algorithms were used to calculate the long-range electrostatic interactions, constrain hydrogen bonds and restrict water molecules, respectively [88,93]. Pymol 1.3.X was further used to generate visual conformation.

### 3.11. Statistical Analysis

All experiments were carried out in triplicate, and the data were presented as mean ± standard deviations (SD). Statistical analysis was performed using analysis of variance (ANOVA) and Tukey’s HSD test with GraphPad Prism 9.0 (GraphPad Software, San Diego, CA, USA). * *p* < 0.05, ** *p* < 0.01 and *** *p* < 0.001 were considered as statistically significant, highly significant and very highly significant, respectively.

## 4. Conclusions

In summary, Myr mainly inhibited glycation of BSA at the end stage. Additionally, Myr suppressed the formation of amyloid-like fibrils and the products of protein glycoxidation, illustrating its strong anti-glycoxidation ability. Moreover, spectroscopic results showed that Myr could quench the endogenous fluorescence of BSA through the static quenching mechanisms. The thermodynamic parameters proved that the binding process was spontaneous and driven by hydrogen bonding and van der Waals forces for Myr-BSA. The binding of Myr and BSA led to slight conformational and microenvironmental changes of BSA as studied by synchronous fluorescence spectroscopy and UV-*vis* spectra. Furthermore, molecular docking revealed that Myr interacted with several residues Lys187, Arg256 and Arg198 of BSA, which prevented the glycation of BSA from sugars and avoided the misfolding and structural transition of BSA. The displacement experiments indicated that Myr primarily bound to site 1 (subdomain IIA) of BSA. These findings revealed the potential antiglycation activity of Myr and the interaction mechanism between Myr and BSA and might provide a strategy for the management of AGEs related diseases.

## Figures and Tables

**Table 1:** Fluorescence parameters of the BSA-Myr system.

Parameters	298 K	308 K
*K_sv_* (10^5^ mol/L)	1.21 ± 0.01	0.41 ± 0.01
*K_q_* (10^13^ L/mol/s)	1.21 ± 0.01	0.41 ± 0.01
*R* ^2*a*^	0.989	0.996
*n*	1.50 ± 0.04	1.35 ± 0.12
*K_a_* (10^4^ mol/L)	3.79 ± 0.18	1.93 ± 0.27
*R* ^2*b*^	0.999	0.987
*ΔG* (kJ/mol)	−26.12 ± 0.01	−24.45 ± 0.01
*ΔH* (kJ/mol)	−51.53 ± 0.58	−51.53 ± 0.58
*ΔS* (J/mol/K)	−85.25 ± 2.38	−87.91 ± 2.36

**Table 2:** Related thermodynamic parameters of probe displacement.

System	*K′_a_* (10^4^ mol/L)	*K′_a_/K_a_* (%)	*R* ^2^
BSA-Myr	3.79 ± 0.18	-	0.999
[BSA + Met]-Myr	1.79 ± 0.22	45.00 ± 4.12	0.994
[BSA + War]-Myr	0.98 ± 0.15	25.76 ± 3.06	0.984
[BSA + Ibu]-Myr	3.38 ± 0.33	89.23 ± 4.81	0.994

**Table 3:** The excitation and emission wavelength of different fluorescent AGEs.

Different AGEs	Excitation Wavelength (nm)	Emission Wavelength (nm)
Total AGEs	350	440
Vesperlysine	350	405
Crossline	380	440
Argpyrimidine	320	380
Pentosidine	335	385

## Data Availability

Not applicable.

## Abbreviations

Myr	Myriocin
AG	Aminoguanidine
BSA	Bovine serum albumin
AGEs	Advanced glycation end products
SPT	Serine palmitoyltransferase
NBT	Nitroblue tetrazolium
ThT	Thioflavin T
Trp	Tryptophan
Tyr	Tyrosine
Phe	Phenylalanine
*ΔG*	Gibbs free energy change
*ΔH*	Enthalpy change
*ΔS*	Entropy change
*λex*	Excitation wavelength
*λem*	Emission wavelength
MD	Molecular dynamics
RMSD	Root mean square deviation
RMSF	Root mean square fluctuation
Rg	Radius of gyration
